# A physiological increase in insulin suppresses muscle‐specific ubiquitin ligase gene activation in fetal sheep with sustained hypoglycemia

**DOI:** 10.14814/phy2.12045

**Published:** 2014-06-24

**Authors:** Laura D. Brown, Stephanie R. Thorn, Meghan C. O'Meara, Jinny R. Lavezzi, Paul J. Rozance

**Affiliations:** 1 Perinatal Research Center, Department of Pediatrics, University of Colorado Denver, Aurora, Colorado, USA; 2 Center for Women's Health Research, University of Colorado Denver, University of Colorado School of Medicine, Aurora, Colorado, USA

**Keywords:** Autophagy‐Lysosome, MaFBx1, MuRF1, pregnancy, ubiquitin‐proteosome

## Abstract

Decreased glucose transfer to the fetus is characteristic of pregnancies complicated by maternal under nutrition and placental insufficiency. Chronic experimental restriction of glucose transfer to the sheep fetus for the final 40% of gestation with a maternal insulin infusion (HG fetuses) results in fetal hypoglycemia, hypoinsulinemia, and decreased rates of fetal growth and protein accretion compared to controls (CON). Lower rates of fetal protein accretion are due to increased fetal protein breakdown and not decreased protein synthesis. However, the specific skeletal muscle pathways responsible for increased protein breakdown have not been determined. Nor has it been determined if low fetal glucose or insulin concentrations are more important for regulating these skeletal muscle protein breakdown pathways. We tested whether chronic restriction of glucose transfer to the fetus increased the ubiquitin–proteosome pathway or autophagy‐lysosome pathway in fetal sheep skeletal muscle and found no evidence for an increase in the autophagy‐lysosome pathway. However, HG fetuses had increase mRNA expression of MaFBx1 (twofold, *P *<**0.01) and a trend for increased mRNA expression of MuRF1 (*P *=**0.08) compared to CON. A subset of chronically hypoglycemic fetuses received an isoglycemic insulin infusion for the final 7 days of the maternal insulin infusion (HG + INS fetuses) and had MaFBx1 and MuRF1 mRNA concentrations similar to CON fetuses. These results demonstrate that fetuses exposed to sustained hypoglycemia have decreased protein accretion due to activation of the skeletal muscle ubiquitin–proteosome pathway and that a fetal hyperinsulinemic clamp can suppress this pathway even in the context of continued hypoglycemia.

## Abstract (precis)

This study shows the importance of the ubiquitin–proteosome pathway for regulating protein accretion during chronic fetal hypoglycemia. Furthermore, we show that this pathway can be suppressed by physiological concentrations for fetal insulin.

## Introduction

Decreased glucose transfer to the fetus is characteristic of pregnancies complicated by maternal under nutrition and placental insufficiency, both of which result in intrauterine growth restriction (IUGR) (Chandler et al. 1985; Limesand et al. 2007). In addition to reduced glucose transfer to the fetus, placental transfer of oxygen and amino acids also may be decreased (Brown et al. 2012; Ganguly et al. 2012). Fetal endocrine responses to nutrient and oxygen deprivation include decreased circulating plasma insulin and IGF‐1 concentrations and increased circulating norepinephrine concentrations (Oliver et al. 1993; Brown et al. 2012; Ganguly et al. 2012). All of these factors contribute to decreased lean mass accretion in the IUGR fetus, including reductions in skeletal muscle growth (Hill et al. 1989; Padoan et al. 2004; Zhu et al. 2004; Larciprete et al. 2005; Belkacemi et al. 2010; Brown et al. 2012).

To determine the specific role of chronically reduced glucose supply on fetal skeletal muscle growth, we have developed a model of restricted placental transfer of glucose to the fetus over the final 40% of gestation in sheep (HG model) (Carver et al. 1997; Thorn et al. 2012; Lavezzi et al. 2013). These fetuses are hypoglycemic, hypoinsulinemic, and have low circulating insulin‐like growth factor‐1 (IGF‐1) concentrations. However, uterine and umbilical blood flows are maintained at normal rates and placental‐to‐fetal transfer rates of oxygen and amino acids are normal, as are circulating fetal norepinephrine concentrations (Narkewicz et al. 1993; Carver and Hay 1995; Carver et al. 1997; Thorn et al. 2012; Lavezzi et al. 2013). Thus, the HG model provides a unique capacity to test the specific effects of chronic fetal hypoglycemia and reduced growth factor availability on fetal skeletal muscle growth, independent of other common features of placental insufficiency, such as hypoxemia and reduced umbilical blood flow.

Previous studies have shown lower fetal protein accretion rates after fetal hypoglycemia was sustained during the last 40% of gestation in the HG model (Carver et al. 1997). Lower protein accretion rates in the HG model were the result of increased protein breakdown rates, as opposed to decreased fetal protein synthesis, as demonstrated using stable isotope methodology to measure amino acid kinetics (Carver et al. 1997). However, the mechanisms by which chronic restrictions in fetal glucose supply increased fetal protein breakdown were not determined. In adults, catabolic states such as starvation, cancer, and burn injury activate proteolytic pathways in skeletal muscle for supplying amino acids for utilization by the heart, liver, and brain (Biolo et al. 1995, 2002; Kadar et al. 2000). Two proteolytic systems are active within skeletal muscle: the ubiquitin–proteosome pathway (mediated by ubiquitin ligases F‐box‐only protein 32 [*FBXO32*; Atrogin ‐1 or MaFBx1] and muscle RING‐finger protein‐1 [*MURF1*]) and the autophagy‐lysosome pathway. These pathways are under coordinated control with protein synthetic pathways to maintain proper cell size to match the available substrate supply (Bonaldo and Sandri 2013). They are activated in muscle when amino acids are required for utilization by vital organs during periods of stress such as trauma, cancer, diabetes, and renal failure (Lecker et al. 2004; Pedroso et al. 2012).

Our aims for this study were twofold. First, we used the HG model to test the hypothesis that chronic restriction of placental glucose transfer to the fetus would activate skeletal muscle protein breakdown pathways. We focused on key regulators of ubiquitin–mediated proteolysis and genes that regulate cellular autophagy. Second, we determined whether the resulting hypoglycemia or hypoinsulinemia produced in the HG model was the primary regulator of skeletal muscle protein breakdown pathways. To test this second aim, we restored fetal insulin concentrations to high physiological levels at the end of the chronic HG period, for the final week of the 8‐week experimental period, without correcting fetal glucose concentrations.

## Materials and Methods

### Animal preparation and experimental design

Studies were conducted in pregnant Columbia‐Rambouillet sheep, each carrying a singleton fetus, in compliance with the Institutional Animal Care and Use Committee, University of Colorado at the Perinatal Research Center in Aurora, CO. This laboratory is accredited by the National Institutes of Health, the United States Department of Agriculture, and the American Association for Accreditation of Laboratory Animal Care. Physiological data, including nutrient, oxygen, and hormone concentrations from these animals, have been previously published (Thorn et al. 2012; Lavezzi et al. 2013). Physiological data relevant for the interpretation of study are shown in Table 1. An initial surgery was performed in 14 pregnant ewes at 70.0 ± 0.8 dGA to place maternal catheters (Thorn et al. 2012). One randomly assigned group of pregnant ewes (*n *=**9) received a continuous maternal infusion of intravenous insulin for 8 weeks to produce hypoglycemic fetuses. Maternal arterial plasma glucose was measured at least twice daily and the insulin infusion was adjusted to achieve a 40–50% reduction in glucose concentrations. The control group received a maternal saline infusion at rates matched to the insulin infusion rates (CON; *n *=**5). At 119.4 ± 0.5 dGA, a second surgery was performed to place fetal catheters in all animals. After the second surgery, hypoglycemic ewes were further randomly divided into two groups. Fetuses in one of these groups received a direct fetal insulin infusion for the final week of the study to produce a fourfold physiologic increase in plasma insulin concentration relative to HG (50% increase vs. CON) (HG + INS; *n *=**4) (Green et al. 2011). The insulin infusion was kept constant at 100 mU/h (using necropsy weights = 38.9 ± 2.8 mU/kg/h) and ran concurrently with a direct fetal infusion of 33% dextrose (wt/vol) to prevent a further fall in fetal glucose concentrations while maintaining high physiological insulin concentrations (Thorn et al. 2012). Fetal arterial plasma glucose concentrations were measured at least twice daily and the dextrose infusion was adjusted accordingly. The remaining hypoglycemic fetuses received a direct fetal saline infusion matched at equal infusion rates to the combined insulin and dextrose infusion (HG; *n *=**5). Fetuses in the 8‐week CON group also received a direct fetal saline infusion at equal rates. Fetal arterial plasma was sampled at the end of the infusions for glucose, insulin, and oxygen concentrations (Thorn et al. 2012; Lavezzi et al. 2013).

**Table 1.** Maternal and fetal arterial parameters and growth characteristics.

	Control	Hypoglycemic	HG + INS
Maternal plasma glucose (mg/dL)	73.9 ± 3.3	32.3 ± 3.5***	32.3 ± 2.7***
Fetal			
Plasma glucose (mg/dL)	22.1 ± 2.0	8.7 ± 0.9***	7.6 ± 0.3***
Plasma insulin (ng/mL)	0.46 ± 0.09	0.16 ± 0.03*	0.66 ± 0.17#
Plasma IGF‐1 (ng/mL)	30.4 ± 6.7	13.2 ± 1.8*	16.5 ± 3.1*
Plasma norepinephrine (pg/mL)	464.3 ± 18.9	549.3 ± 158.7	843.1 ± 177.2
Oxygen content (mmol/L)	3.58 ± 0.12	3.22 ± 0.26*	1.75 ± 0.59* #
Gestational age (days)	133.2 ± 1.1	133.0 ± 1.3	134.5 ± 0.9
Total weight (gm)	3662 ± 122	2204 ± 137**	2611 ± 214**
Carcass weight (gm)	2754 ± 97	1654 ± 110**	1662 ± 33.8**
Crown‐rump length (cm)	49.2 ± 1.3	43.5 ± 0.6*	43.0 ± 1.2*
Lower limb length (cm)	35.3 ± 1.2	30.1 ± 1.4*	29.9 ± 1.3*

Data from the end of the study are shown as means ± SE for control (*n *=**5), hypoglycemic (*n *=**5), and hypoglycemic + insulin (HG + INS, *n *=**4).

*, **, *** refer to *P* values <0.05, 0.001, and 0.0001 versus control. # refers to *P* value <0.05 versus HG.

### Biochemical analysis

Whole blood was collected in EDTA coated syringes and immediately centrifuged (14,000 g) for 3 min at 4°C. The plasma was stored at −70°C for hormone measurements. Insulin, IGF‐1, and norepinephrine concentrations were measured as previously described (Thorn et al. 2012; Lavezzi et al. 2013). Oxygen content of the blood was immediately measured on whole blood collected in heparin coated syringes with the ABL 520 analyzer (Radiometer, Copenhagen, Denmark). Samples were then centrifuged for 60 sec and plasma was removed for glucose concentration measurement using the YSI model 2700 select biochemistry analyzer (Yellow Springs Instruments, Yellow Springs, OH) (Thorn et al. 2012; Lavezzi et al. 2013).

### Skeletal muscle analysis

Skeletal muscle from the biceps femoris was collected and snap‐frozen in liquid nitrogen and stored at −70°C until further analysis.

#### mRNA expression

For mRNA analysis, total RNA was extracted from pulverized skeletal muscle (200 mg) and reverse transcribed into complimentary DNA (cDNA), as described previously (Limesand et al. 2007). Ovine F‐box‐only protein 32 (*FBXO32*; Atrogin ‐1 or MaFBx1), ring finger protein 28 (*RFP28*; MuRF1), heat shock protein kDa protein 5 (*HSPA5*; glucose regulatory protein 78, grp78), *β*‐actin (*ACTB*) and Glyceraldehyde 3‐phosphate dehydrogenase (*GAPDH*) were measured using real‐time quantitative PCR (qPCR) as previously described (Rozance et al. 2007; Limesand et al. 2009; Gadhia et al. 2013). DNA damage‐inducible transcript 3 (*DDIT3*; C/EBP homologous protein, CHOP), class III phosphatidyl inositol 3‐kinase (*PIK3C3*; vacuolar protein sorting 34, Vps34), BCL2/adenovirus E1B 19 kDa protein‐interacting protein (*BNIP3*), cathepsin L1 (*CTSL1*), beclin‐1 (*BECN1*), and activating transcription factor 4 (*ATF4*) were identified in ovine muscle cDNA and primers for real‐time qPCR developed and optimized as previously described (Rozance et al. 2007). Specificity of the primers (Table 2) for the target genes was confirmed with agarose gel electrophoresis, melting curve analysis, and sequencing of the PCR products. cDNA samples were run in triplicate, and the qPCR was performed as described previously (Limesand et al. 2009), with the standard curve method of relative quantification used to compare results (Wong and Medrano 2005). GAPDH and actin were used as housekeeping genes and were not different between treatment groups.

**Table 2.** Gene primers and amplicon size.

Gene	Forward	Reverse	Amplicon size
*FBXO32*	AAA GTC CTT GAA GAC CAG CAA	AAG CAC AAA GGC AGG TCT GT	232
*RFP28*	TGT GCC AAC GAC ATC TTC CA	GAT GAT GTT CTC CAC CAG CA	168
*HSPA5*	TGA AAC TGT GGG AGG TGT CA	GCA GGA GGA ATT CCA GTC AG	191
*DDIT3*	CAC TCT TGA CCC TGC CTC TC	GCT CGA TTT CCT GTT TGA GC	277
*ACTB*	TGC AGA AAG AGA TCA CTG CC	GAC AGC GAG GCA GGA TGG	110
*GAPDH*	TGG AGG GAC TTA TGA CCA CTG	TAG AAG CAG GGA TGA TGT TCT	120
*PIK3C3*	GAG ACC CAA AGA CCC ATG AAA	CCA GCA GAG AAC GCA TAA CT	102
*MAP1LC3A*	TAA AGA GGT GCA GCA GAT CC	ACC AAC TCG CTC ATG TTG AC	141
*BNIP3*	TGC GCA ACA CGA GCG TCAT	CGT TGT CAG GCG CCT TCC	133
*CTSL1*	CCT GTG AAG AAT CAG GGT CA	ATT AGG CCA CCA TTG CAA CC	166
*BECN1*	CAG GAG GAA GCT CAG TATC A	CAG CTT GTC CAG CTG CATC	122
*ATF4*	CAT GGG TTC TCC TGC GAC A	TCT CCA CCA TCC AAT CTG TC	124

#### Protein expression

Protein was extracted from pulverized skeletal muscle (100 mg) and quantified as previously described (Limesand et al. 2009). Twenty microgram of protein were separated by polyacrylamide gel electrophoresis under reduced conditions (5% *β*‐mercaptoethanol). Proteins were then transferred to a nitrocellulose or polyvinylidene difluoride membrane (BioRad, Hercules, CA). Western blotting was performed as previously described (Brown et al. 2009, 2012; Limesand et al. 2009; Thorn et al. 2009, 2012). All membranes were blocked for one hour in PBS with 0.01% tween 20 (PBST; BioRad) and 5% nonfat dried milk (NFDM; w/v) for one hour at room temperature, except membranes probed for insulin receptor *β* (IR*β*) which were blocked with PBST and 5% NFDM (w/v) plus 1% bovine serum albumin (BSA; w/v). The primary antibody for IR*β* (1:1250, Santa Cruz Biotechnology, Santa Cruz, CA) also was diluted in this buffer. The following primary antibodies were diluted in PBST with 5% BSA (w/v): micorotubule‐associated proteins 1A/1B light chain 3A (LC3; 1:1000, Cell Signaling Technology Inc., Danvers, MA), S235/236 phosphorylated ribosomal protein S6 (rpS6; 1:500, Cell Signaling), rpS6 (1:2000, Cell Signaling), T421/S424 phosphorylated p70 S6 kinase (P70S6K; 1:500, Cell Signaling), P70S6K (1:500, Cell Signaling), T37/46 phosphorylated eukaryotic Initiation Factor 4E binding protein 1 (4E‐BP1, 1:2000, Cell Signaling), 4E‐BP1 (1:500, Cell Signaling), S52 phosphorylated eukaryotic initiation factor 2*α* (eIF2*α*, 1:3000, Stressgen, Ann Arbor, MI), eIF2*α* (1:1000, Stressgen), T56 phosphorylated eukaryotic elongation factor 2 (eEF2, 1:2500, Cell Signaling), eEF2 (1:2500, Cell Signaling), S473 phosphorylated protein kinase B (PKB or Akt; 1:2000, Cell Signaling), Akt (1:2000, Cell Signaling), T202/Y204 phosphorylated extracellular regulated kinase 1/2 (ERK 1/2, 1:1000, Cell Signaling), ERK 1/2 (1:5000, Cell Signaling), T172 adenosine monophosphate activated kinase (AMPK; 1:500, Cell Signaling), and AMPK (1:750, Cell Signaling). The following primary antibodies were diluted in PBST with 5% NFDM (w/v): the p85 subunit of the phosphotidyl inositol 3 kinase (p85; 1:10,000, Upstate, Billerica, MA) and actin (1:50,000; MP Biomedicals, Santa Ana, CA). Horseradish peroxidase (HRP) conjugated secondary antibodies were diluted in PBST with 5% NFDM and applied to membranes for one hour at room temperature and then washed. Immunocomplexes were detected with enhanced chemiluminescence (Amersham ECL Plus, Piscataway, NJ). Densitometry was performed using Scion Image software (Scion Corporation, Frederick, MD). All results were normalized to *β*‐actin to control for loading differences. Phosphorylated specific immunoblots are presented as the ratio of the phosphorylated amount to the total amount of each respective protein. Antibodies were stripped from the membranes with Restore Western Stripping Buffer (Pierce, Rockford, IL).

### Statistical analysis

Statistical analysis was performed using SAS version 9.1 (Cary, NC). All results are presented as mean ± standard error. Previously published physiological data obtained at multiple time points were analyzed with mixed models ANOVA which included terms for experimental group, time, and a group by time interaction. A term for repeated measures obtained from the same animal was included. Skeletal muscle protein, mRNA, and glycogen measurements were analyzed by one‐way ANOVA. A *P* value of ≤0.05 was considered significant. Individual means were compared using Fisher's Least Significant Differences Test.

## Results

### Maternal and fetal characteristics

In vivo data and growth characteristics have been reported and are summarized here (Table 1) (Thorn et al. 2012). Maternal arterial plasma glucose concentrations in the HG and HG + INS group were lower compared to those in the CON group throughout the study (*P *<**0.0001, Table 1), but did not differ from each other. Fetuses in the HG group were hypoglycemic (*P *<**0.0001) and hypoinsulinemic (*P *<**0.05) compared to fetuses in the CON group (Table 1). HG + INS fetuses were infused for the final 7 days with exogenous insulin and glucose to maintain a 1 week fetal hyperinsulinemic‐hypoglycemia clamp (Table 1). Fetal arterial blood oxygen content was similar between CON and HG fetuses, but was nearly 50% lower in HG + INS (*P *<**0.05). Fetal arterial norepinephrine concentrations were similar between CON and HG and were variably increased in HG + INS. Fetal arterial IGF‐1 concentrations were lower in both HG and HG + INS compared to CON (Table 1). Despite similar gestational ages, HG and HG + INS fetuses weighed significantly less and had significantly lower carcass weights than CON fetuses (*P *<**0.001). Furthermore, both total fetal and lower limb lengths were less in HG and HG + INS fetuses compared to CON fetuses (*P *<**0.05, Table 1).

### Ubiquitin–proteosome pathway

**Figure 1.**
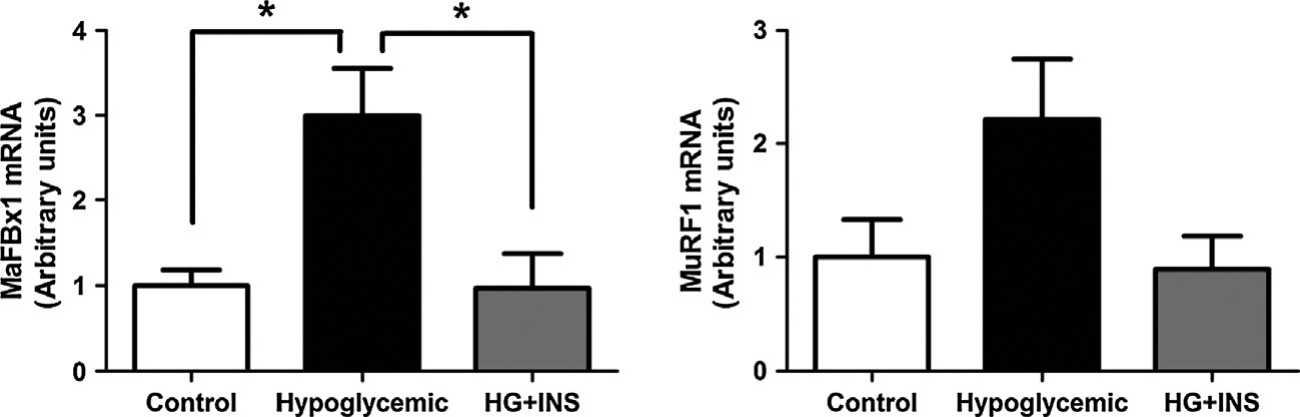
Effect of hypoglycemia on skeletal muscle ubiquitin ligase gene expression. MaFBx1 and MuRG1 mRNA expression was measured by real‐time qPCR in the late gestation fetal biceps femoris skeletal muscle from control (*n *=**5), hypoglycemic (*n *=**5), and hypoglycemic + insulin (HG + INS, *n *=**4). *Indicates *P *<**0.01. Overall ANOVA for MuRF1 *P *=**0.08.

Fetal biceps femoris mRNA expression of two muscle specific ubiquitin ligases was measured. MaFBx1 was twofold higher in the HG fetuses compared to CON (*P *<**0.01, Fig. 1). Expression in HG + INS fetuses was significantly lower than in HG fetuses (*P *<**0.01) and not different from CON (Fig. 1). MuRF1 followed a similar pattern (*P *=**0.08, Fig. 1).

### Autophagy‐lysosome pathway

**Figure 2.**
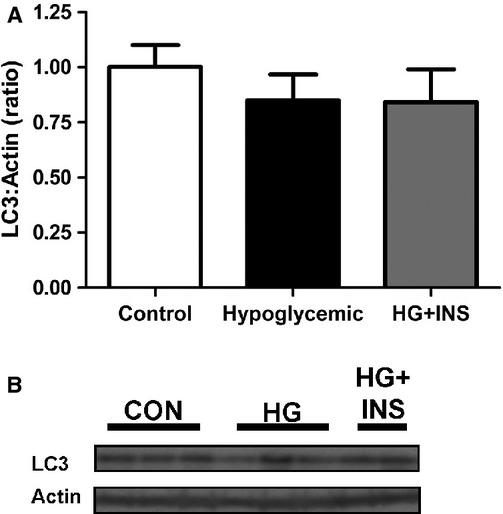
Effect of hypoglycemia on LC3. LC3 protein expression was measured by western blotting in the late gestation fetal biceps femoris skeletal muscle from control (*n *=**5), hypoglycemic (*n *=**5), and hypoglycemic + insulin (HG + INS,* n *=**4). (A) Densitometry quantification. (B) Representative western blot.

Given the increase in ubiquitin ligases, we next measured components of the autophagy‐lysosome pathway. Protein expression of LC3, a marker for autophagosome formation (Mizushima and Yoshimori 2007), was similar among the groups (Fig. 2). Furthermore, the mRNA expression of genes associated with cellular autophagy (*BNIP3*, Beclin 1, Cathepsin L1, and Vps34) was similar among the groups (Table 3).

**Table 3.** mRNA expression.

Gene	Control	Hypoglycemic	HG + INS
Autophogy‐lysosome pathway			
BNIP3	1.00 ± 0.11	1.07 ± 0.17	1.09 ± 0.19
Beclin‐1	1.00 ± 0.17	1.06 ± 0.25	0.72 ± 0.08
Cathepsin L1	1.00 ± 0.15	1.25 ± 0.26	0.80 ± 0.09
Vsp34	1.00 ± 0.25	1.90 ± 0.45	0.92 ± 0.22
Nutrient sensing			
CHOP	1.00 ± 0.20	1.67 ± 0.55	1.18 ± 0.27
GRP78	1.00 ± 0.15	1.36 ± 0.42	0.93 ± 0.16
ATF4	1.00 ± 0.15	0.97 ± 0.20	0.73 ± 0.07

Values are mean ± SE for control (*n *=**5), hypoglycemic (*n *=**5), and hypoglycemic + insulin (HG + INS, *n *=**4), and are normalized to expression in the control group.

### Protein synthesis pathway

**Figure 3.**
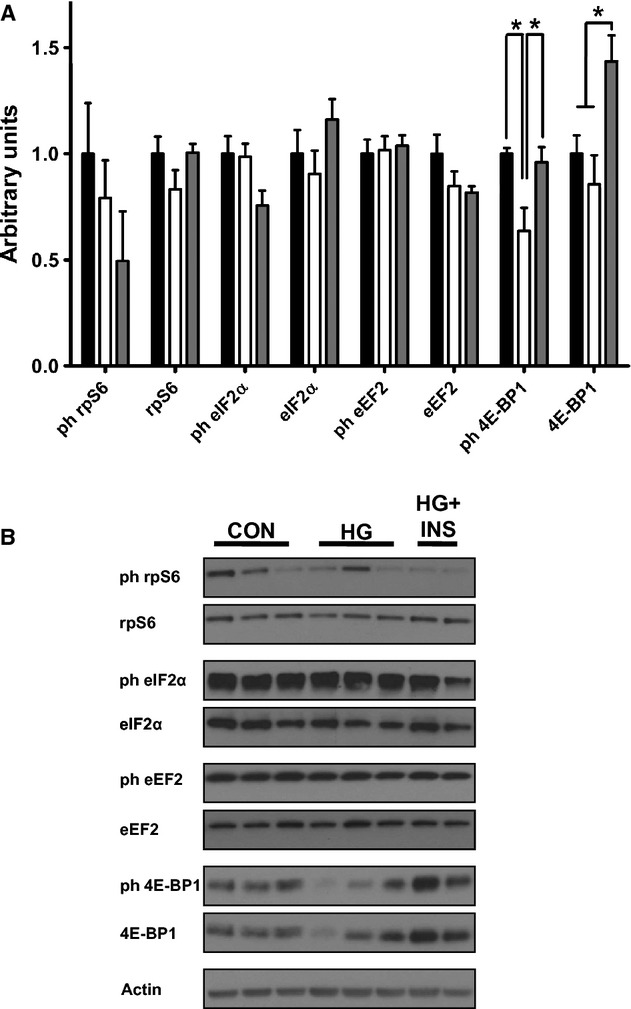
Effect of hypoglycemia on skeletal muscle mRNA translation factors. Protein expression of mRNA translation factors was measured by western blotting in the late gestation fetal biceps femoris skeletal muscle from control (*n *=**5), hypoglycemic (*n *=**5), and hypoglycemic + insulin (HG + INS,* n *=**4). (A) Densitometry quantification, *Indicates *P *<**0.05. (B) Representative western blots.

To determine if there were differences in protein synthetic pathways, we measured expression and activation of signaling proteins that regulate mRNA translation initiation and protein elongation. There was less phosphorylated 4E‐BP1 in the HG group (*P *<**0.01), which was restored with 1 week of insulin infusion in the HG + INS group (*P *<**0.05, Fig. 3). The total amount of 4E‐BP1 also was increased in the HG + INS group (*P *<**0.01, Fig. 3). There were no differences among CON, HG and HG + INS groups for the phosphorylated or total amounts of mRNA translation initiation and elongation factors eEF2*α*, eIF2*α*, and rpS6.

### Nutrient sensing and insulin signaling pathways

**Figure 4.**
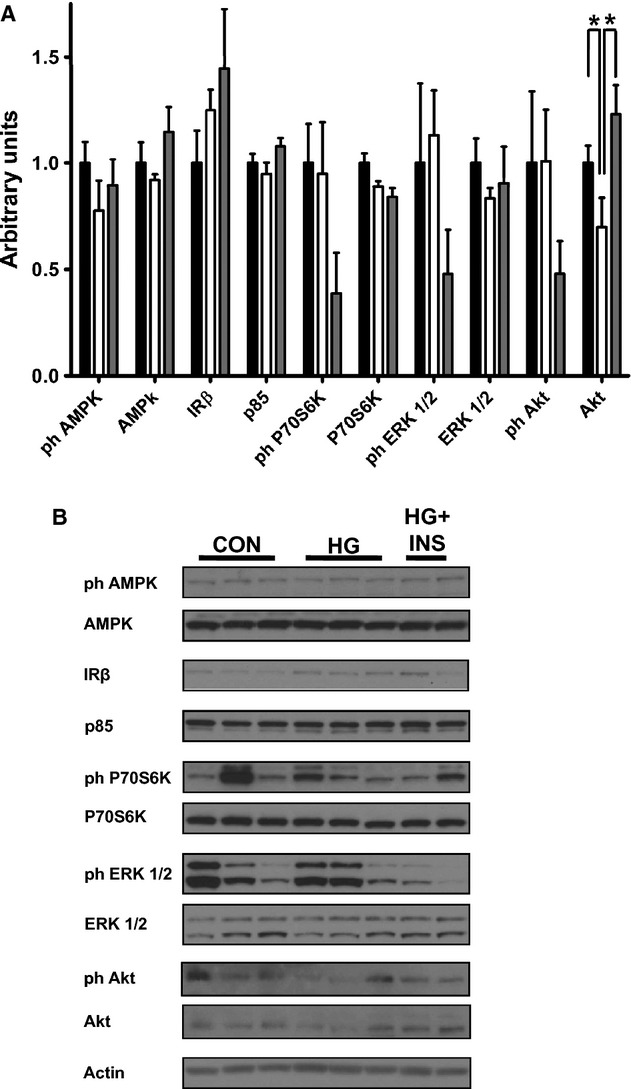
Effect of hypoglycemia on skeletal muscle nutrient sensors and insulin signaling kinases. Protein expression of nutrient sensors and insulin signaling kinases was measured by western blotting in the late gestation fetal biceps femoris skeletal muscle from control (*n *=**5), hypoglycemic (*n *=**5), and hypoglycemic + insulin (HG + INS,* n *=**4). (A) Densitometry quantification, *Indicates *P *<**0.05. (B) Representative western blots.

Protein and mRNA expression of several cellular nutrient sensors, which regulate proteolysis and protein synthesis, were not different among the three groups, including protein expression of total and phosphorylated AMPK (Fig. 4) and mRNA expression of GRP78, CHOP, and ATF4 (Table 3).

Several insulin signaling proteins that regulate cellular proliferation and protein synthesis, including the insulin receptor, the p85 subunit of PI3K, P70S6K, and ERK, were not significantly different among the groups (Fig. 4). Although there appeared to be less total Akt in the HG group, this did not reach statistical significance (*P *=**0.09), but Akt expression increased in the HG + INS group compared to the HG group (*P *<**0.05). Although there appeared to be less phosphorylated Akt, ERK, and P70S6K in the HG + INS group, these failed to reach statistical significance (*P *=**0.21, *P *=**0.09, *P *=**0.14, respectively, Fig. 4).

## Discussion

In this study, we show that the ubiquitin–proteosome pathway is increased in fetal skeletal muscle in HG fetuses as demonstrated by an increase in MaFBx1 and MuRF1 gene expression. This provides evidence for a mechanism responsible for increased fetal protein breakdown rates found in previous studies (Carver et al. 1997). We found no evidence for upregulation of the autophagy‐lysosome pathway in fetal skeletal muscle. Furthermore, an experimental physiological increase in insulin concentrations for the final week of chronic fetal hypoglycemia resulted in normalization of the expression of the ubiquitin ligases. We did not find any decreases in mRNA translation initiation and elongation factors that would be expected to cause a decrease in the rate of new protein synthesis, also consistent with prior data showing normal rates of protein synthesis in this model (Carver et al. 1997).

MaFBx1 and MuRF1 are muscle‐specific ubiquitin ligases that mediate protein ubiquination and eventual degradation. They are increased in a variety of experimental conditions in adults which result in skeletal muscle atrophy, including denervation, immobilization, underweighting and systemic illness (Bodine et al. 2001; Lecker et al. 2004; Gumucio and Mendias 2013). The role of ubiquitin‐mediated proteolysis in the growing fetus has been less well characterized. One study found increased MaFBx1 expression associated with impaired fetal skeletal muscle growth after exposure of pregnant rats to glucocorticoids and inadequate food intake (Gokulakrishnan et al. 2012). Our laboratory also has previously found increased MaFBx1 and MuRF1 along with increased rates of protein breakdown after 2 weeks of fetal hypoglycemia and hypoinsulinemia (Limesand et al. 2009). Collectively, these data support a role for increased ubiquitin ligase activation and protein breakdown that results after 2–8 weeks of hypoglycemia in the fetus.

We also show for the first time that the increase in ubiquitin ligase expression is reversed by a direct insulin infusion into the hypoglycemic fetuses for the final week of the study. We infused dextrose to prevent a further fall in fetal glucose concentrations, a condition that likely resulted in increased skeletal muscle glucose uptake and utilization. Therefore, suppression of ubiquitin ligase expression with isoglycemic restoration of fetal insulin concentrations indicates that fetal insulin concentrations or increased rates of fetal myocellular glucose metabolism regulate skeletal muscle ubiquitin ligases and protein degradation to a greater extent than fetal glucose concentrations alone. While in vitro skeletal muscle cell studies and in vivo human adult data support a direct role for insulin independent of glucose, a 1‐week fetal hyperglycemic clamp with a concurrent somatostatin infusion to block an associated increase in fetal insulin concentrations in chronic HG fetuses is required to differentiate these possibilities (Tesseraud et al. 2007; Dalbo et al. 2013). IGF‐1 also suppresses ubiquitin ligase expression in cultured myotubes (Sandri et al. 2004). Interestingly, HG and HG + INS fetuses both have lower IGF‐1 concentrations than CON, supporting a greater role for insulin, rather than IGF‐1, in normalizing ubiquitin ligase expression in the HG + INS group.

There were other systemic effects of the chronic insulin infusion, including lower arterial oxygen and reductions in essential branched chain amino acid concentrations (BCAAs; leucine, isoleucine, and valine) in HG + INS compared to the HG fetuses (Lavezzi et al. 2013). Low oxygen has been shown to decrease fetal protein accretion in the fetus, but this was due to a lower rate of new protein synthesis and not an increase in protein breakdown (Milley 1987, 1998). Decreased oxygen and amino acids, especially the BCAAs, would be expected to decrease muscle protein accretion and lead to an increase in skeletal muscle ubiquitin ligases and autophagy activation (Milley 1994; Herningtyas et al. 2008; Sugawara et al. 2009; Baptista et al. 2010; Maki et al. 2012; de Theije et al. 2013; Masschelein et al. 2014; Suryawan and Davis 2014). Therefore, despite lower fetal oxygen and BCAAs, hyperinsulinemia in the HG + INS fetuses reduces and normalizes skeletal muscle ubiquitin ligase expression. We cannot exclude effects of other nutrients, nutrient metabolism and hormones on muscle protein breakdown pathways which were not measured in this study.

Chronic restriction of placental glucose transfer to the fetus does not result in decreased rates of synthesis of new protein (Carver et al. 1997; Limesand et al. 2009). Consistent with this, HG fetal skeletal muscle did not demonstrate a pattern of decreased activation of translation initiation or elongation factors that would be expected to inhibit the synthesis of new protein. Among all the factors measured, only a significant decrease in the phosphorylation of 4E‐BP1 was observed. In its phosphorylated state, 4E‐BP1 inhibits the formation of the translation initiation complex. Decreased phosphorylation of 4E‐BP1 in the HG fetuses, if anything, therefore, would increase protein synthesis.

Interestingly, increased insulin concentrations for 1 week did not have a major impact on translation initiation or elongation factors either, although the numbers of animals in each group may have precluded our ability to detect subtle differences in these or other pathways. As expected with insulin stimulation (Shen et al. 2002), the phosphorylation of 4E‐BP1 increased, but so did the total amount of 4E‐BP1. Thus, 4E‐BP1 appears to be the most sensitive to changes in glucose and insulin concentrations in the fetus. However, none of the other factors involved in protein synthesis or insulin signaling changed significantly, which is in contrast with marked stimulation of insulin signaling after acute stimulation of fetal muscle with insulin (Shen et al. 2002; Brown et al. 2009, 2012). The reason for the differences between these results and those for acutely hyperinsulinemic fetuses are not clear. There is time dependency in the insulin responsiveness of each of these factors (Anderson et al. 2005), but there may also be differences between the response of normally growing fetuses and those in this study, whose glucose supply has been chronically restricted. In fact, selective inhibition of downstream components of insulin signaling within skeletal muscle has been observed in fetuses of pregnant ewes that were fasted for 5 days during late gestation (Shen et al. 2005). Thus, substrate availability to the fetus affects the capacity of growth factors to regulate translation initiation in fetal skeletal muscle.

Adenosine monophosphate activated kinase is a cellular nutrient sensor which is phosphorylated and activated during periods of cellular energy insufficiency and activated AMPK increases both the ubiquitin–proteosome and autophagy‐lysosome pathways and decreases protein synthesis pathways in skeletal muscle (Sanchez et al. 2012). Interestingly, skeletal muscle AMPK phosphorylation was unchanged in both the HG and HG + INS fetuses, consistent with other models of chronic fetal nutrient deprivation (Zhu et al. 2004; Thorn et al. 2009, 2012). Therefore, the increase in skeletal muscle ubiquitin ligase expression is independent of the AMPK pathway.

### Perspectives and significance

Prolonged restriction of placental glucose transfer to the fetus for 8 weeks in pregnant sheep made hypoglycemic by maternal insulin infusion resulted in activation of the skeletal muscle ubiquitin ligases MaFBX1 and MuRF1. This occurred in the absence of activation of the autophagy‐lysosome pathway or protein synthesis signaling pathways. Importantly, our results also show that increased expression of the ubiquitin ligases can be prevented or reversed with a prolonged fetal insulin infusion, which argues that availability of this growth factor in the fetus induces skeletal muscle protein ubiquination and degradation more than low glucose concentrations. We speculate that, as in the adult under conditions of nutrient deprivation, lack of growth factor stimulation, or other stressful conditions, fetal skeletal muscle can serve as a reservoir to deliver substrates such as amino acids to maintain metabolism and energy production, but at the expense of lean mass accretion and muscle growth. This provides yet another example of how the fetal metabolism independently adapts to adverse conditions to maintain fetal survival to the end of gestation.

## Acknowledgments

We thank Karen Trembler, David Caprio, Alex Cheung and Gates Roe for their technical support. The authors have no conflicts of interest to disclose.

## Conflict of Interest

None declared.
